# The Convergent Frontier: Integrating Molecular, Computational, and Surgical Sciences in Oncology

**DOI:** 10.3390/biomedicines13081983

**Published:** 2025-08-15

**Authors:** Aya Hasan Alshammari, Takaaki Hirotsu, Hideyuki Hatakeyama, Eric di Luccio

**Affiliations:** Hirotsu Bioscience Inc., New Otani Garden Court 22F, 4-1 Kioi-cho, Chiyoda-ku, Tokyo 102-0094, Japan; hirotsu@hbio.jp (T.H.); h.hatakeyama@hbio.jp (H.H.); e.diluccio@hbio.jp (E.d.L.)

## 1. Introduction

The long-standing pillars of oncology—surgery, molecular biology, and computational science—are no longer advancing in parallel; they are converging into a single, powerful force. It is the central argument of this editorial that the next generation of breakthroughs in cancer care will be driven by this synergistic integration. This second edition of the Special Issue on “Advanced Cancer Diagnosis and Treatment” serves as a testament to this paradigm shift, assembling eleven outstanding articles that map the landscape of this new, unified ecosystem. Spanning from bench-side molecular discovery [1,2] and the challenges of rare diseases [3,4] to the data-driven quantification of surgical practice [5,6] and the deployment of AI in clinical decision-making [7,8,9]. Together, they present a compelling vision of a future where multidisciplinary insights are woven into a unified approach to patient care.

## 2. AI and Computational Advances: From Diagnosis to Surveillance

A central theme of this Special Issue is the maturation of artificial intelligence from discrete applications into an integrated capability spanning the entire oncological care continuum. The contributions herein illustrate this trajectory, demonstrating AI’s impact from initial diagnosis and staging through to therapeutic planning and long-term surveillance. At the crucial juncture of diagnosis, Sarkar et al. pioneer a transfer learning framework using a pre-trained ResNet-18 model to classify lymph node metastases in prostate cancer [8].

By outperforming classical radiomic algorithms, this approach shows how deep learning can overcome the challenge of limited datasets to enhance staging accuracy. In the domain of prognostication, another contribution introduces a sophisticated, interpretable algorithm to predict relapse risk for patients after resection of colorectal cancer liver metastases [7]. This model, which synergizes logistic regression with machine learning, provides clinicians with a transparent, evidence-based tool to guide decisions on adjuvant therapy.

Finally, Buga et al. address post-treatment surveillance by developing a deep learning model to monitor brain metastases after Gamma Knife radiosurgery [9]. Their framework, demonstrating exceptionally high classification accuracy (AUC = 1.0), is seamlessly integrated into a clinical decision support application with real-time predictions and visualizations for model interpretability.

Collectively, these studies [7,8,9] offer a powerful narrative of AI’s evolution from a niche application to an essential component of precision medicine, delivering continuity and foresight throughout the patient’s journey.

## 3. Evidence-Based Quantification of Surgical Oncology

Surgical oncology is undergoing a critical evolution from a practice based on qualitative principles to a paradigm of quantitative, evidence-based, and risk-adapted decision-making. The articles in this issue illustrate this shift by addressing two complementary facets of surgical risk management: selecting the right candidates for intervention and technically optimizing the procedure. First, addressing patient selection, a 20-year analysis of liver transplantation for hepatocellular carcinoma (HCC) provides a robust empirical foundation for risk modeling [5]. By quantifying key predictors of recurrence, such as preoperative alpha-fetoprotein (AFP) levels and microvascular invasion, this work enables more precise identification of patients who will benefit most from this resource-intensive therapy. Second, highlighting procedural optimization, Steffens et al. deliver an evidence-based directive on surgical margin width in high-grade soft tissue sarcomas [6]. Their research establishes that a resection margin of >5 mm is a crucial determinant for superior local control and survival, providing a quantifiable and actionable surgical target to mitigate recurrence risk. Together, these studies [5,6] showcase the maturation of surgical science, where rigorous data analysis is leveraged to optimize decisions both before and during an operation, transforming clinical art into a data-driven science.

## 4. Diagnostic Innovation: Resolving Clinical and Therapeutic Ambiguity

Modern diagnostics increasingly serve to resolve the clinical and therapeutic ambiguities that confound cancer management. The contributions highlighted here exemplify this theme by tackling uncertainty at multiple scales. At the macroscopic level, Sökeland et al. directly confront diagnostic ambiguity in poorly differentiated liver masses where standard histomorphology is inconclusive [10]. Their proposed workflow, which integrates immunohistochemistry with molecular analysis for TERT promoter mutations, provides decisive diagnostic clarity and informs appropriate management. At the cellular level, Simsone et al. address the profound ambiguity of therapeutic failure by exploring its fundamental mechanisms [1]. Through detailed characterization of paclitaxel-treated melanoma cells, they identify a drug-tolerant subpopulation of “microcells” that exhibit stem-like properties, offering a mechanistic explanation for chemoresistance. Further upstream, a comprehensive review of the Limbic System-Associated Membrane Protein (LSAMP) establishes its role as a tumor suppressor whose loss is linked to metastasis across numerous cancers [2]. This work identifies LSAMP as a promising pan-cancer biomarker, adding a crucial piece to the molecular puzzle of tumorigenesis. These investigations [1,2,10] demonstrate the power of moving beyond traditional pathology, using molecular genetics to resolve diagnostic uncertainty and decipher the mechanisms of progression and resistance.

## 5. Navigating the Complexity of Rare Oncological Diseases

Beyond common malignancies, this Special Issue also illuminates the distinct challenges posed by rare cancers, where limited data and diagnostic ambiguity demand specialized investigation. A comprehensive review of rosette-forming glioneuronal tumors (RGNTs) provides a vital resource for understanding this rare CNS entity, detailing its clinical, pathological, and molecular landscape [4]. This work highlights how molecular characterization is key to resolving diagnostic complexity in uncommon tumors. The challenge of building an evidence base for rare diseases is addressed by a retrospective multicenter analysis of primary renal lymphoma (PRL), another rare entity [3]. By pooling data from 32 cases, the study identifies crucial prognostic factors and provides evidence supporting the role of nephrectomy, demonstrating how collaborative efforts are essential to establish standards of care where large trials are not feasible. These papers [3,4] underscore the critical importance of integrating molecular data and fostering multicenter collaboration to build a robust evidence base for diagnosing and treating rare malignancies, ensuring these patient populations are not left behind.

## 6. Advanced Imaging and Fluorescence-Guided Therapy

The real-time visualization of neoplastic tissue during resection constitutes a significant clinical advance, aimed at maximizing cytoreduction while preserving healthy tissue. This principle is powerfully substantiated by a systematic review and meta-analysis, which provides Level 1 evidence for the clinical superiority of 5-aminolevulinic acid (5-ALA) fluorescence-guided surgery (FGS) in high-grade gliomas [11]. The analysis confirms that 5-ALA FGS results in significantly higher rates of gross total resection and confers a definitive advantage in both progression-free and overall survival compared to conventional white-light surgery. This robust evidence not only solidifies its position as a standard of care but also underscores the clinical value of integrating real-time biological data into the surgical workflow.

## 7. Concluding Remarks: A Call for Integrated Oncology

In conclusion, the research gathered in this Special Issue powerfully illustrates that multidisciplinary collaboration is the engine of oncological progress. The works presented herein argue that the future of oncology lies not in the advancement of siloed technologies but in their thoughtful and synergistic integration. The central challenge for the scientific community, therefore, is to build a unified clinical ecosystem: one where AI-driven insights are rigorously validated and integrated into practice; where molecular biomarkers from tissue and liquid biopsies guide therapeutic decisions in real-time; and where advanced surgical and imaging techniques become equitably accessible to all patients. We are confident that this collection will serve as both an essential reference and a catalyst, inspiring the research community to pursue these vital integrative goals and ultimately translate today’s cutting-edge science into tomorrow’s standard of care.
